# Associations Between Physical Stimulus Size and Vertical Response Locations: Small Goes Down and Large Goes Up

**DOI:** 10.1027/1618-3169/a000655

**Published:** 2026-02-10

**Authors:** Peter Wühr, Oliver Lindemann

**Affiliations:** ^1^ Department of Psychology, TU Dortmund University, Germany; ^2^ Erasmus University Rotterdam, the Netherlands

**Keywords:** horizontal, metaphor, size–hand compatibility, size–location compatibility, SSARC effect, vertical

## Abstract

**Abstract:** This study investigates the existence and direction of associations between physical stimulus size and vertical response locations. In Experiment 1, 80 participants responded to stimulus size by pressing one of two vertically arranged keys. We orthogonally manipulated within-subjects the mappings between stimulus size and response-key locations, and between hands and response keys. The results showed a novel compatibility effect between physical stimulus size and vertical response location: Responses to the small stimulus were faster at the lower than at the higher location, whereas responses to the large stimulus were faster at the higher than at the lower location. In Experiment 2, we replicated this compatibility effect in vocal responses, when 66 participants responded to stimulus size by saying location words. In combination with previous findings, the present results suggest that the physical size of visual objects is not only associated with horizontal locations but also with vertical locations. These associations presumably reflect learned correlations between the size of objects and their extension in both the horizontal and the vertical direction. Moreover, the observation of size–space compatibility effects in vocal responses may indicate that they do not only reflect sensorimotor experiences but also semantic knowledge, which is expressed in linguistic metaphors.


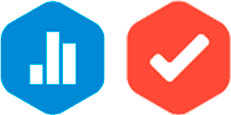


Research has identified associations between the cognitive representations of feature dimensions that would appear unrelated at first sight. These associations are often expressed in so-called compatibility or congruency effects (e.g., Alluisi & Warm, 1990; Kornblum & Lee, 1995). The so-called *spatial-numerical association of response codes* (SNARC) effect is a well-known example for a congruency effect between two apparently unrelated feature dimensions, namely, between numerical size and space. The SNARC effect refers to the finding of faster and more often accurate left-hand responses to numerically small relative to large numbers, and faster and more often accurate right-hand responses to numerically large relative to small numbers (e.g., Dehaene et al., 1993; see Fischer & Shaki, 2014, for review). The SNARC effect has been interpreted as evidence for structural overlap between the cognitive representations of numerical size and space (e.g., Dehaene et al., 1993). Several authors have described strong associations between numbers and space by using the metaphor of a mental number line, where numbers are represented in the ascending order from left to right (e.g., Dehaene et al., 1993; Hubbard et al., 2005). Importantly, the compatibility effect between numerical magnitudes and spatial responses can be attributed to a shared, generalized system for processing analog magnitude-related information across different domains (Sixtus et al., 2023; Walsh, 2003). This common cognitive metric is believed to underlie the encoding of all size-related information, whether it is physical, spatial, temporal, or numerical.

Motivated by an embodied view of cognition, Winter et al. (2015) argued that spatial–numerical associations are not limited to the horizontal dimension but also extend to the vertical and sagittal dimensions. Empirical support for vertical associations has been shown in studies on mental arithmetic (e.g., Wiemers et al., 2014), in random number generation tasks (e.g., Winter & Matlock, 2013), and in the parity-judgment task (e.g., Aleotti et al., 2020; Ito & Hatta, 2004). In contrast, however, studies using number-classification tasks with vertically arranged manual responses produced inconclusive results (Hartmann et al., 2014; but see Wiemers et al., 2017a).

The present study addresses the so-called Spatial-Size Association of Response Codes (SSARC) effect,^1^ a novel instance of a compatibility effect involving two conceptually distinct feature dimensions – namely, physical size and spatial response location. The SSARC effect refers to the finding of faster and more often accurate left-hand responses to physically small relative to large stimuli, and faster and more often accurate right-hand responses to physically large relative to small stimuli (e.g., Ren et al., 2011; Wühr & Seegelke, 2018). The SSARC effect provides empirical evidence for a structural overlap between the cognitive representations of physical size and space (e.g., Wühr & Seegelke, 2018). Although both physical size and space do not only vary along the horizontal dimension, the SSARC has hitherto only been investigated for the horizontal dimension, that is, with responses that vary along the horizontal dimension. Therefore, the main purpose of the present study is to investigate, for the first time, whether the SSARC effect extends to the vertical dimension, that is, whether compatibility effects between physical stimulus size and spatial responses also emerge when response alternatives are arranged along the vertical dimension. In the following, we will describe some characteristics of the horizontal SSARC effect and two possible accounts for this effect. Then, we will describe other compatibility effects with vertically arranged responses and discuss arguments for and against the existence of a vertical SSARC effect.

## Characteristics of the Horizontal SSARC Effect

In a typical task for investigating the SSARC effect, participants are instructed to press a left- or right-hand key to the physical size of a visual stimulus (e.g., 2 vs. 4 cm of size), and the size-response mapping is varied between blocks. Several studies showed faster and more often accurate responses for the compatible stimulus response mappings (i.e., “small – left, large – right”) than for the incompatible mappings (i.e., “small – right, large – left”; Seegelke et al., 2023; Wühr & Seegelke, 2018, Experiment 1). Notably, the relationship between stimulus size and response location still affects performance when stimulus size is irrelevant for the task at hand (i.e., congruency effect).^2^ In a typical task, participants are instructed to press a left- or right-hand key to a physical stimulus feature other than size, such as color or shape, and stimulus size is varied independently from the relevant stimulus feature. The results show faster and more often accurate left responses when the stimulus is small, and faster and more often accurate right responses to color or shape when the stimulus is large (e.g., Wühr & Seegelke, 2018, Experiment 2; Richter & Wühr, 2022). The presence of these S-R congruency effects suggests that participants unintentionally encode stimulus size, and the resulting representation of stimulus size automatically activates a congruent response (e.g., Wühr & Heuer, 2024; Wühr & Seegelke, 2018).

Recent studies suggest that the physical stimulus size is encoded in a context-specific manner, that is, relative to the elements of the stimulus set in a given task. Wühr and Richter (2022) used two different stimulus sets, which overlapped in an intermediate-sized stimulus (i.e., set of smaller stimuli: 1, 2, and 3 cm; set of larger stimuli: 3, 4, and 5 cm), in different blocks of their experiment. When participants responded to the color (Experiment 1) or shape (Experiment 2) of the stimuli, comparable SSARC effects were observed across both stimulus sets, demonstrating a relative and context-dependent coding of stimulus size. This is particularly evident with regard to the stimulus size shared by both sets (i.e., size 3). This size represented the largest element in the set of smaller stimuli, but the smallest element in the set of larger stimuli. Participants responded faster with their left hand than with their right hand to stimulus 3 when it was part of the smaller set, whereas the opposite pattern was observed when the same stimulus was part of the larger set. Similar support for a highly context-dependent congruency effect has been reported for spatial associations of numerical magnitudes (SNARC effect, e.g., Ben Nathan et al., 2009; Dehaene et al., 1993; Fischer, 2006). A recent study suggests, however, that SNARC effects are not fully modulated by the context and that certain effects of the spatial associations with the absolute magnitude persist even under different contexts and different SNARC effects with relative magnitude. (Roth, et al., 2025).

Other studies investigated the spatial characteristics of the responses that are relevant for the SSARC effect. Several results of these studies suggest that using the hands as effectors, or the requirement to choose between the hands for responding, plays a special role for the SSARC effect. For example, the SSARC effect only occurs in tasks with manual responses when response selection requires a choice between the left or right hand, while the effect is absent when response selection requires a choice between two adjacent fingers of the same hand (Seegelke et al., 2023). Notably, effects of spatial S-R correspondence (e.g., Heister et al., 1987) and the SNARC effect (e.g., Priftis, et al., 2006) still occur when response selection requires a choice between two fingers of the same hand, which suggests that adjacent fingers are coded as left or right according to their current location in space (see also Brozzoli et al., 2008). Moreover, the participants handedness affects the size of the SSARC effect, which is another finding that suggests a special role of the two hands for the SSARC effect. In particular, the SSARC effect (i.e., the superiority of the small-left/large-right mapping over the small-right/large-left mapping) is larger for right-handers relative to left-handers (Wühr et al., 2024). Again, this finding contrasts with the finding that handedness does not seem to affect the SNARC (e.g., Cipora et al., 2019; Dehaene et al., 1993).

## Explanations of the Horizontal SSARC Effect

There are, at least, two viable explanations for the horizontal SSARC effect, the polarity-correspondence account, and the correlations-in-experience (CORE) account. The polarity-correspondence principle assumes that participants unintentionally assign polarities to the stimuli and responses in many binary classification tasks (Proctor & Cho, 2006; Proctor & Xiong, 2015). With regard to the SNARC effect, Proctor and colleagues proposed that negative polarity is assigned to small numbers and to left responses, whereas positive polarity is assigned to large numbers and to right responses (e.g., Proctor & Cho, 2006). Response selection is assumed to be impaired (i.e., delayed) when there is a mismatch between the polarities of the stimulus and the response in a given trial (e.g., small-right, large-left) relative to when the polarities match (e.g., small-left, large-right). The SNARC effect is attributed to this impairment of response selection in case of noncorresponding polarities (e.g., Proctor & Cho, 2006; Proctor & Xiong, 2015).

The polarity-correspondence account of the SNARC effect can also be applied to the SSARC effect. With regard to the latter, one would assume that negative polarity is assigned to physically small objects and left responses, whereas positive polarity is assigned to physically large objects and right responses (e.g., Richter & Wühr, 2022). This relationship may be rooted in the fact that more is often better than less in everyday life. Again, better performance is expected when the polarities of a stimulus and a response match in a given trial than when the polarities mismatch. The polarity-correspondence account is consistent with several characteristics of the SSARC effect such as, for example, the finding that relative stimulus size, and not absolute stimulus size, is responsible for the SSARC effect (Wühr & Richter, 2022).

Other characteristics of the SSARC effect are, however, inconsistent with a polarity-correspondence account. For example, the failure to observe the SSARC effect when response selection requires a choice between two adjacent fingers of the same hand (Seegelke, et al., 2023) is inconsistent with the polarity-correspondence account. This finding strikingly contrasts with the fact that both spatial S-R congruency effects (e.g., Heister et al., 1987) and the SNARC effect (e.g., Priftis et al., 2006) do occur in tasks that require a response choice between two fingers. Importantly, the observation of spatial S-R congruency and SNARC effects with choices between two fingers of the same hand strongly suggests that two adjacent fingers of the same hand are coded as “left” versus “right,” and hence, the preconditions for polarity correspondence are present in this condition as well.

The correlations-in-experience (CORE) principle (Pitt & Casasanto, 2020) provides another possible explanation for the SSARC effect. The CORE principle is part of the “hierarchical mental metaphors” (HMMT) theory (e.g., Casasanto, 2017) and has originally been proposed to account for the SNARC effect. HMMT assumes that people use concrete (i.e., directly observable) feature dimensions – called “source domains” – for thinking and talking about more abstract feature dimensions – called “target domains” (e.g., Casasanto, 2017; see also Lakoff & Johnson, 1980). For example, people use spatial terms and spatial relationships for thinking and talking about time. These mental metaphors are not only reflected in verbal metaphors (e.g., “it’s high time”) but also in compatibility/congruency effects between time and space (e.g., Gevers et al., 2003; see Umiltà et al., 2018, for review). According to the CORE principle, mental metaphors are learned by observing correlations between source and target domains. These correlations can be registered through bodily experiences, but also through language, cultural habits, or artifacts (cf. Casasanto, 2017; Pitt & Casasanto, 2020). In an attempt to explain the origin of the (horizontal) SNARC effect, Pitt and Casasanto (2020) identified finger-counting habits as an important source for providing correlations between numerical size and spatial location. These authors demonstrated that the direction of finger counting (from left to right or from right to left) in a short practice session can modulate the size of the SNARC effect in a subsequent test, providing empirical evidence for the CORE principle as an account of SNARC. Note, however, that the results of other studies cast doubts on the relationship between finger-counting habits and the SNARC, at least, when natural counting behavior instead of instructed behavior is considered (e.g., Hohol et al., 2022).

The CORE principle implies that different compatibility or congruency effects result from different experiences of correlations between source and target domains. That is, a correlation between numerical size and horizontal location in finger counting may explain the SNARC effect, but it cannot explain the SSARC effect. Recently, Wühr et al. (2024) proposed that the SSARC effect may have originated from a correlation between hand strength and object size. The right hand is the dominant hand in most people, and the dominant hand is known to be stronger than the nondominant hand (e.g., Bardo et al., 2021; see Foley et al., 2025, for a review). Wühr et al. hypothesized that the strength difference between dominant and nondominant hand may have led to the habit of grasping larger (and heavier) objects with the dominant hand and grasping smaller (and lighter) objects with the nondominant hand. The resulting correlation between object size and hand (side) may have eventually led to the SSARC effect.

Wühr et al. (2024) provided empirical evidence for their hypothesis. First, they showed that handedness modulates the size of the SSARC effect: The effect is larger in right-handers than in left-handers. Second, the researchers also observed that strength differences between the two hands correlated with the size of the SSARC effect (in Reaction Times, RTs): The stronger the right hand relative to the left hand, the larger the SSARC effect as measured by the difference in RTs between the compatible and the incompatible mapping.

## S-R Compatibility With Vertically Arranged Responses

Until now, SSARC effects have exclusively been shown with horizontally arranged responses, indicating that small objects are more strongly associated with left responses, and large objects are more strongly associated with right responses, than vice versa. The goal of the present study is to investigate whether SSARC effects can also occur with vertically arranged responses and, if this was the case, what the dominant associations between stimulus size and vertical locations are.

The existence of vertical SSARC effects can be expected for several reasons. First, many S-R compatibility effects are not restricted to horizontal S-R arrangements, but occur for vertical S-R arrangements as well. For example, spatial S-R compatibility effects do not only occur with horizontal S-R arrangements (e.g., Brebner, 1973; Wallace, 1971), but also occur with vertical S-R arrangements (e.g., Nicoletti & Umiltà, 1984; Hedge & Marsh, 1975).

Second, the size of most objects in the real world typically covaries on three dimensions. Therefore, one might expect strong associations between “small” and “down,” and between “large” and “up,” confirming a “more is up” metaphor (Lakoff, 1993; Lakoff & Johnson, 1980). Lakoff (1993, p. 240) characterizes this metaphor as follows “the MORE IS UP metaphor is grounded in experience – in the common experiences of pouring more fluid into a container and seeing the level go up, or adding more things to a pile and seeing the pile get higher.” In the context of this view, the size of an object is a fundamental example for a perceived continuous magnitude and should thus activate spatial association. A vertical SSARC effect would thus reflect sensorimotor experiences as, for example, one may touch and grasp a larger (i.e., taller) object at a higher place than a smaller (i.e., lower) object.

Spatial–numerical associations have also been investigated with S-R compatibility effects and vertically arranged responses, but these studies have produced inconsistent, and sometimes surprising, findings. For example, Hartmann et al. (2014) compared vertical SNARC effects in tasks in which participants pressed vertically arranged keys either with the two hands or with hand and foot. The researchers observed a “more is up” SNARC effect when participants operated the keys with different hands, whereas they observed a “more is down” SNARC effect when participants operated the upper key with a hand and the lower key with a foot. The reason for the reversal of the vertical SNARC effect with hand and foot as effectors remained open.

In a more recent study, Wiemers et al. (2017a) investigated SNARC effects with vertically arranged response keys, but independently varied the stimulus-key mapping (small-down, large-up vs. small-up, large-down), and the hand-key mapping (left-down, right-up vs. left-up, right-down). In two experiments, in which the hand-key mapping changed only once in the middle of the experiment, Wiemers et al. observed only effects of the hand-key mapping, with left-down/right-up producing superior performance than left-up/right-down, but no vertical SNARC effect. A vertical SNARC effect, that is, an effect of stimulus-key mapping was only observed in Experiment 3, when the hand-key mapping changed repeatedly after each block of trials. These results suggest that the associations between numerical size and hands are stronger than those between numerical size and vertical locations.

As mentioned above, both the SNARC and SSARC effect represent instances of the association between spatial and size-related information, yet they can be explained by different underlying mechanisms. For example, as shown by Wiemers et al. (2017b), horizontal spatial–numerical associations and spatial associations of physical size occur simultaneously but independently of each other, suggesting they have different cognitive origins. Moreover, independent of whether the SNARC and SSARC effects are the result of a common system for analogue magnitudes (Walsh, 2003), polarity correspondence (Proctor & Cho, 2006), or experiencing correlations between space and size (Pitt & Casasanto, 2020), the origin of the two effects is likely to differ, because acquired number concepts – in contrast to physical size – comprise not only a cardinal meaning (i.e., magnitude information) but also have ordinal meaning (see, e.g., Sixtus et al. 2023). Research on serial order processing and the SNARC effect suggests that numerical associations with space are dynamically constructed in working memory (Lindemann et al., 2008; van Dijck & Fias, 2011) and stem predominantly from the number's ordinal position rather than its cardinal meaning. Considering this aspect, the SNARC effect might have a different origin than the SSARC effect.

In the following, we report two experiments that addressed the existence and the direction of SSARC effects between the size of stimuli and vertically arranged responses. In Experiment 1, we investigated SSARC effects between physical stimulus size and the vertical position of two response keys that were operated with the two hands. In Experiment 2, we then investigated the existence and direction of SSARC effects between the physical size of stimuli and the spoken responses “above” and “below.”

## Experiment 1

In Experiment 1, we investigated the existence and direction of SSARC effects between the physical size of stimuli and the vertical locations of response keys that are operated by the two hands. In accordance with the study on the vertical SNARC effect by Wiemers et al. (2017a), we independently varied (a) the mapping between stimulus size and vertical key position and (b) the mapping between vertical key position and the operating hand. If the SSARC effect behaves like the SNARC effect (e.g., Wiemers et al., 2017a), we should observe a hand-related SSARC effect between stimulus size and responding hand, but we should fail to observe a location-related SSARC effect between stimulus size and vertical key location. This result would also be consistent with findings suggesting a special role of the two hands for the occurrence of the SSARC effect (e.g., Seegelke et al., 2023; Wühr et al., 2024). On the other hand, however, positive correlations between the width and height of objects can often be observed in the world, for example, when objects are moving toward or away from us. In addition, sensorimotor experiences might also differ between objects of different height. For example, we may touch and grasp larger (i.e., taller) objects at higher locations than smaller (i.e., lower) objects. Such correlations might be learned, and subsequently lead to SSARC effects between stimulus size and vertical locations.

### Method

Experiment 1 was pre-registered at OSF (https://doi.org/10.17605/OSF.IO/9NYQ7) on October 16, 2023.

#### Openness and Transparency

The local Ethics Committee at TU Dortmund University (2018–09) approved the experimental protocol for both experiments. The data from both experiments are available at Mendeley (https://doi.org/10.17632/8jw866px99.2). Moreover, we report how we determined our sample size, data exclusion, all manipulations, and all measures in the study.

#### Participants

In a previous study with a large sample (N = 160), we observed a strong horizontal SSARC effect in *manual* responses with partial η^2^ = .31 (Wühr et al., 2024). Since we are lacking studies on vertical SSARC effects, we cut this effect size by half, which gives us partial η^2^ = .155. A sample size of 74 participants is required to detect a 2 × 2 interaction of this size with high power (1-β = .95) at the standard .05 α error probability.

The actual sample consisted of 80 participants, mostly students at TU Dortmund University. The participants had an average age of 22.48 years (*SD* = 4.38), and consisted of 56 females and 24 males. According to self-report, all participants had normal or corrected-to-normal vision, and 72 participants were right-handers. Participants gave their informed consent prior to participation and received course credits or a coffee voucher (€ 2.50) for compensation.

#### Apparatus and Stimuli

**Figure 1:**
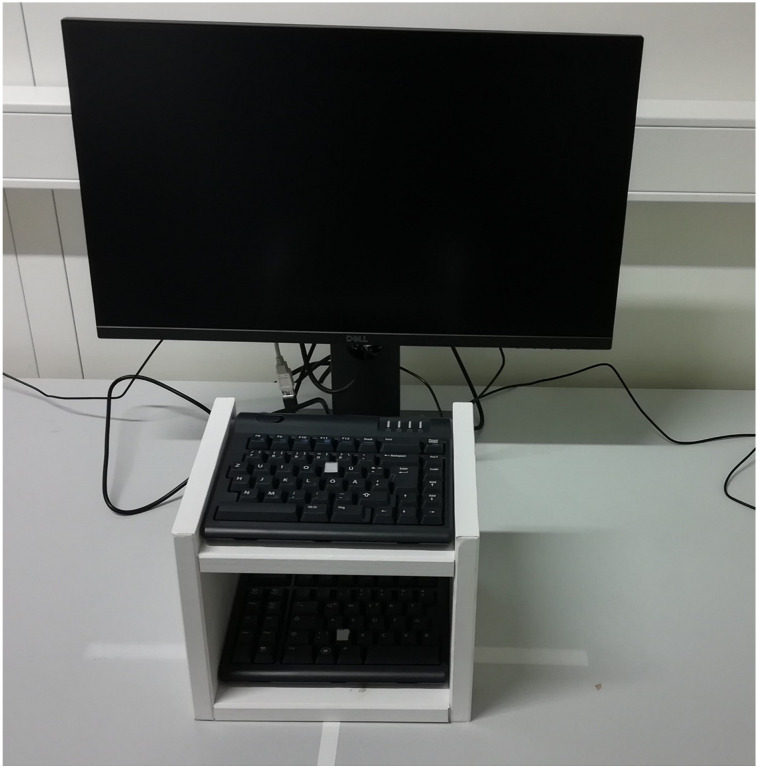
Response apparatus used for Experiment 1.

Participants sat in front of a 24-inch monitor with a viewing distance of 50 cm. We used the software E-Prime 3.0 (Psychology Software Tools; Sharpsburg, PA, USA) to control the presentation of stimuli and register responses. A plus sign (Courier font, size 18 pt) served as a fixation. The imperative stimulus was a black square that varied in size. In particular, the square was either small (i.e., 2 × 2 cm) or large (i.e., 4 × 4 cm). The fixation point and the imperative stimulus were presented on a gray background (E-Prime color ‘silver’, RGB = 192, 192, 192) at the screen center. Participants responded manually by pressing keys that differed in vertical location. Therefore, we used a keyboard that consisted of two parts, and positioned the “left” part of the keyboard on the lower board of a custom-made, wooden rack, and the “right” part of the keyboard in the upper board of the rack (see Figure 1). The lower key was the ‘z’ key, whereas the upper key was the ‘p’ key on this keyboard. The vertical distance between the two keys was approximately 16 cm. The two relevant keys were marked with a rough tape that could be felt with the fingertips.

#### Procedure

There were four conditions in the experiment that differed in (a) the hand-key assignment and (b) the stimulus-key mapping. At the beginning of a condition, the first slide informed participants about the correct hand-key assignment. For one hand-key assignment, participants were told to operate the lower key with the index finger of the left hand and the upper key with the index finger of the right hand. For the other hand-key assignment, participants were told to operate the lower key with the index finger of the right hand and the upper key with the index finger of left hand. Then, instructions informed participants about the relevant stimulus-key mapping for the upcoming condition. With one stimulus-key mapping, participants had to press the lower key to the small stimulus and the upper key to the large stimulus. For the other stimulus-key mapping, participants had to press the upper key to the small stimulus and the lower key to the large stimulus. The four conditions resulted from combining two hand-key assignments with two stimulus-key mappings.

Each condition included one training block consisting of 20 trials and two experimental blocks consisting of 40 trials each. Trials occurred in a random order within each block. Both imperative stimuli occurred with equal frequency. Each trial started with a blank screen of 500 ms. Next, the fixation point was presented for 400 or 600 ms at the screen center, with both durations occurring equally often within a block.^3^ Then, the imperative stimulus occurred and remained until a response was made or for 2,000 ms. Each response was followed by an intertrial interval of 1,000 ms with an empty screen. If an incorrect key was pressed, or if no response was detected during the presence of the imperative stimulus, a corresponding error message was shown during the intertrial interval.^4^

We counterbalanced the order of conditions across participants as follows. Half of the participants made the two stimulus-key mapping conditions first with the down-left/up-right hand-key assignment, and then with the down-right/up-left hand-key assignment. The other half of the participants had the opposite order. Moreover, for both orders of the hand-key assignments, half of the participants faced the small-down/large-up mapping before the small-up/large-down mapping, whereas the other half of the participants faced the two mapping conditions in the reverse order. Participants could take a short break between blocks. For each condition, the experimenter stayed in the laboratory for the practice blocks and left the laboratory before participants started the first experimental block. The experiment took about 30 min.

#### Design and Data Analysis

The experiment had a three-factorial 2 × 2 × 2 design with *Stimulus Size* (small, large), *Response Hand* (left, right), and *Response Location* (above, below) as within-subjects variables.^5^ Dependent variables were the RTs of correct keypress responses, and the percentages of incorrect responses.

We excluded training blocks, the first trial in each experimental block, and trials in which RTs were below 100 ms or above 1,500 ms from data analysis. An outlier screening using the Tukey criterion (i.e., 1.5 × IQR – Quartile_25_; 1.5 × IQR + Quartile_75_) identified three outlier values in overall RT and three outlier values in overall error percentage. However, no participant showed outlier performance in both dependent variables simultaneously, and therefore, we refrained from excluding participants from the analysis. Excluding the six participants would not affect the qualitative pattern of results.

### Results

#### RTs

**Figure 2:**
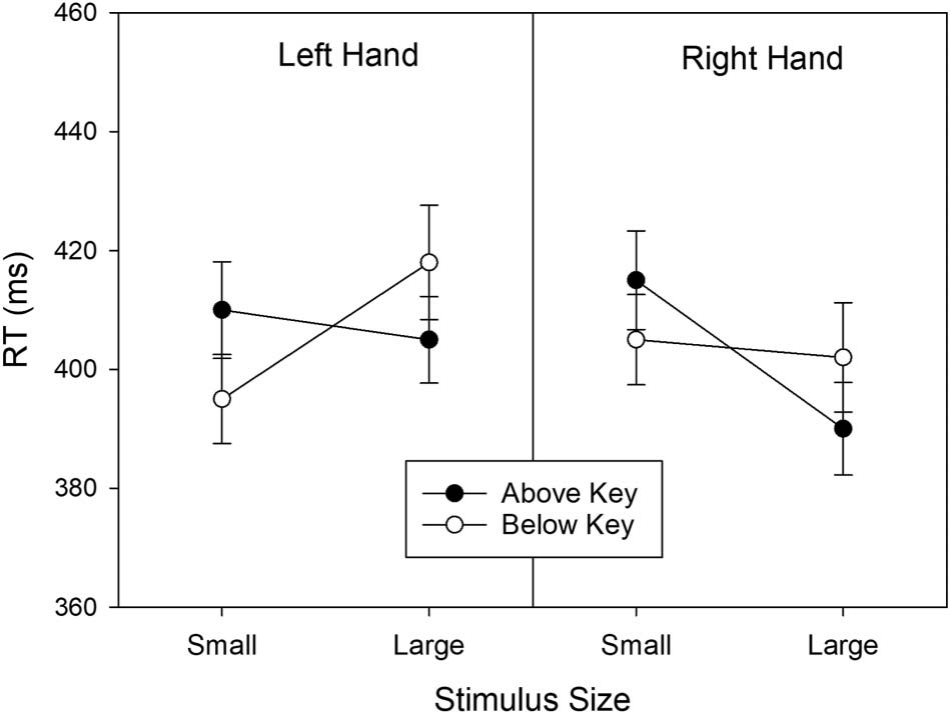
Mean RTs observed in Experiment 1 as a function of stimulus size, response hand, and response-key location. Error bars represent 95% confidence intervals for within-subjects designs (Cousineau, 2017).

We conducted a three-factorial ANOVA with Stimulus Size, Response Hand, and Response Location as within-subject factors, and RT as the dependent variable. The means for the eight cells of this design are presented in Figure 2. An almost significant main effect of Response Hand, *F*(1, 79) = 3.937, *MSE* = 705.504, *p* = .051, partial η^2^ = .047, reflected shorter right-hand RTs (*M* = 403 ms, *SD* = 53) compared to left-hand RTs (*M* = 407 ms, *SD* = 54). The main effects of Stimulus Size, *F*(1, 79) = 1.495, *MSE* = 718.827, *p* = .225, partial η^2^ = .019, and Response Location, *F*(1, 79) = 0.015, *MSE* = 518.436, *p* = .903, partial η^2^ < .001, were not significant.

A significant two-way interaction of Stimulus Size × Response Hand, *F*(1, 79) = 12.400, *MSE* = 1,618.873, *p* < .001, partial η^2^ = .136, reflected a size-hand congruency effect. In particular, left responses were faster to small stimuli relative to large stimuli, *t*(79) = −2.388, *p* = .019, *d* = −0.267 (two-tailed test), and right responses were faster to large stimuli relative to small stimuli, t(79) = 3.424, *p* < .001, *d* = 0.383 (two-tailed test).^6^ A significant two-way interaction of Stimulus Size × Response Location, *F*(1, 79) = 18.814, *MSE* = 1,364.133, *p* < .001, partial η^2^ = .192, reflected a size-location congruency effect for vertical response locations. In particular, “below” responses were faster to small stimuli relative to large stimuli, *t*(79) = −2.692, *p* = .009, *d* = −0.301 (two-tailed test), and “above” responses were faster to large stimuli relative to small stimuli, *t*(79) = 4.397, *p* < .001, *d* = 0.492 (two-tailed test). Finally, the two-way interaction of Response Hand × Response Location, *F*(1, 79) = 0.075, *MSE* = 2,013.763, *p* = .785, partial η^2^ = .001, and the three-way interaction, *F*(1, 79) = 0.872, *MSE* = 433.480, *p* = .353, partial η^2^ = .011, were not significant.

#### Error Percentages

We conducted a three-factorial ANOVA with Stimulus Size, Response Hand, and Response Location as within-subjects factors and error percentage as the dependent variable. The means for the eight cells of the design are given in Table 1. A significant main effect of Stimulus Size, *F*(1, 79) = 8.014, *MSE* = 7.605, *p* = .006, partial η^2^ = .092, reflected less errors with large stimuli (*M* = 2.250, *SD* = 3.465) relative to small stimuli (*M* = 2.867, *SD* = 3.874). The main effects of Response Hand, *F*(1, 79) = 0.826, *MSE* = 6.254, *p* = .366, partial η^2^ = .010, and Response Location, (1, 79) = 1.574, *MSE* = 5.963, *p* = .213, partial η^2^ = .020, were not significant.

**Table 1:** Error percentages observed in Experiment 1 as a function of stimulus size, response hand, and response location (*SD*s are given in parentheses)

Stimulus size	Left hand		Right hand	
	Below	Above	Below	Above
Small stimulus	2.375 (3.079)	2.531 (3.068)	2.500 (3.795)	4.063 (5.037)
Large stimulus	2.906 (4.680)	2.063 (3.201)	1.969 (2.599)	2.063 (2.997)

A significant two-way interaction of Stimulus Size × Response Location, *F*(1, 79) = 9.268, *MSE* = 6.576, *p* = .003, partial η^2^ = .105, reflected a size-location congruency effect for vertical response locations in error percentages. In particular, “below” responses were more accurate than “above” responses to small stimuli, *t*(79) = −3.139, *p* = .002, *d* = −0.341 (two-tailed test), while the accuracy of “below” and “above” responses to large stimuli did not differ, *t*(79) = 1.311, *p* = .194, *d* = 0.147 (two-tailed test). The two-way interaction of Response Hand × Response Location, *F*(1, 79) = 5.930, *MSE* = 9.263, *p* = .017, partial η^2^ = .070, was also significant. This interaction reflected the finding that right-hand responses were more accurate when pressing the lower key than when pressing the upper key, *t*(79) = −2.992, *p* = .004, *d* = −0.335 (two-tailed test, while a nonsignificant trend in the opposite direction occurred for left-hand responses, *t*(79) = 1.019, *p* = .311, *d* = −0.114 (two-tailed test). The two-way interaction of Stimulus Size × Response Hand, *F*(1, 79) = 3.320, *MSE* = 20.262, *p* = .072, partial η^2^ = .040, and the three-way interaction, *F*(1, 79) = 0.491, *MSE* = 4.472, *p* = .485, partial η^2^ = .006, were not significant.

### Discussion

In Experiment 1, we investigated the existence and direction of SSARC effects between the physical size of stimuli and the vertical locations of response keys that were operated by the two hands. Therefore, we independently varied (a) the mapping between stimulus size and vertical key location, and (b) the mapping between vertical key location and operating hand. We observed the already known compatibility effect between physical stimulus size and hands: Left-hand responses were faster to the small as compared to the large stimulus, and right-hand responses were faster to the large as compared to the small stimulus.

The present results clearly show the existence of hand-related SSARC effects with vertical response-key arrangements, proving they are not limited to horizontal arrangements (e.g., Seegelke et al., 2023; Wühr & Seegelke, 2018). In addition, and in contrast to studies on the vertical SNARC effect (Wiemers et al., 2017a), we also observed a vertical SSARC effect, that is, a compatibility effect between physical stimulus size and vertical key locations, which was independent of hands. Lower-key responses were faster to the small as compared to the large stimulus, and upper-key responses were faster to the large as compared to the small stimulus. A similar, although weaker, pattern was observed in error percentages. These observations are consistent with a “more is up” metaphor.

Experiment 1 investigated associations between physical stimulus size and hands, and between physical stimulus size and vertical key location, and not associations between hands and vertical key location. Yet, since we obtained evidence for associations between size and hands, and between size and vertical location, respectively, one might also expect associations between hands and locations. In particular, given the observed results, one might expect that the left hand is more strongly associated with the lower location, whereas the right hand is more strongly associated with the upper location. The results of Experiment 1, however, do not support this hypothesis. In fact, the Hand × Location interaction was not significant for RTs, whereas the error data showed a pattern in the opposite direction. However, when the results of three participants with outlier values in error percentages (all *M* > 9%) are excluded from the analysis, the unexpected interaction is gone, *F*(1, 76) = 3.02, *MSE* = 3.41, *p* = .086, partial η^2^ = .04. Hence, the large majority of participants showed no sign of a Hand × Location interaction in RTs and error percentages.

## Experiment 2

In Experiment 2, we investigated the existence and direction of SSARC effects between physical stimulus size and the spoken responses “above” and “below.” If the SSARC effect for the vertical dimension requires the use of manual responses (at different vertical locations), then we should fail to observe a vertical SSARC effect with spoken responses. If, however, the vertical SSARC effect not only reflects different sensorimotor experiences of the hands but also reflects semantic knowledge, as expressed in metaphors such as “more is up” (e.g., Lakoff, 1993), we should also observe a vertical SSARC effect with spoken responses.

### Method

Experiment 2 was pre-registered at OSF (https://doi.org/10.17605/OSF.IO/HRWQM) on October 11, 2023.

#### Participants

In a previous study with a large sample (N = 160), we observed a strong horizontal SSARC effect in *vocal* responses with partial η^2^ = .333 (Wühr et al., 2024). Since we are lacking studies on vertical SSARC effects, we cut this effect size by half, which gives us partial η^2^ = .17. A sample size of 66 participants is required to detect a 2 × 2 interaction of this size with high power (1-β = .95) at the standard .05 α error probability.

Our sample consisted of 66 participants, mostly students at TU Dortmund University. The participants had an average age of 21.354 years (*SD* = 2.880), and consisted of 56 females and 10 males. All participants declared having normal or corrected-to-normal vision, and the majority (i.e., 60) of the participants described themselves as right-handed. Participants gave their informed consent prior to participation and received course credits or 5.00 Euro as a compensation.

#### Apparatus and Stimuli

The stimuli, and the conditions of stimulus presentation, were the same as in Experiment 1. However, we used a different device for measuring RTs and registering the vocal responses in Experiment 2. Participants responded vocally by speaking into a microphone, which stood directly in front of them, and was connected to the voice-key of the Chronos console (Psychology Software Tools; Sharpsburg, PA, USA). The voice key measured vocal RTs and stored each vocal response in a sound file. Vocal RTs were defined as the interval between the onset of the imperative stimulus and the onset of the vocal response. The voice-key detected a vocal response when the loudness of the response exceeded a preset threshold. Each recording had a duration of two seconds, beginning with stimulus onset. The accuracy of the stored vocal responses was checked offline by the experimenters.

#### Procedure

The experiment consisted of two conditions of a vocal choice–response task. In both tasks, participants responded to stimulus size by speaking a location word (the German word “unten” for “below,” or the German word “oben” for “above”)^7^ as quickly as possible. In one condition, the S-R mapping required saying “below” to the small stimulus and “above” to the large stimulus, respectively. In the other condition, the S-R mapping required saying “above” to the small stimulus, and “below” to the large stimulus, respectively. Each condition, or task, consisted of a practice block (20 trials) and two test blocks with 40 trials each. Different trials occurred in the random order within each block.

Each trial started with a blank screen of 500 ms. Next, the fixation point was presented for 400 or 600 ms at the screen center, with both durations occurring equally often within a block. Then, the imperative stimulus occurred and remained until a response was made or for 2,000 ms. Each response was followed by an intertrial interval of 1,500 ms with an empty screen. If no response was detected during the presence of the imperative stimulus, a corresponding message (“No response detected”) was shown during the intertrial interval. The program could not determine the accuracy of a spoken response, and therefore, the program did not provide feedback about response accuracy. All other features of the procedure were similar to Experiment 1.

#### Design and Data Analysis

The task had a two-factorial design with Stimulus Size (small, large) and Response (above, below) as within-subjects variables. Dependent variables were the RTs of correct vocal responses and the percentages of incorrect responses.

We excluded training blocks, the first trial in each experimental block, and trials in which RTs were below 100 ms or above 1,500 ms from data analysis. An outlier screening using the Tukey criterion revealed no outliers in overall RT (*RT*_max_ = 612 ms), but six outliers in overall error percentage (*PE*_max_ = 13.125%). Because no participant had both long error rates and long RTs, we did not exclude participants from further analysis. However, we had to drop the data set from one participant because her sound files were of bad quality. Hence, the final sample included 65 participants.

### Results

#### RTs

**Figure 3:**
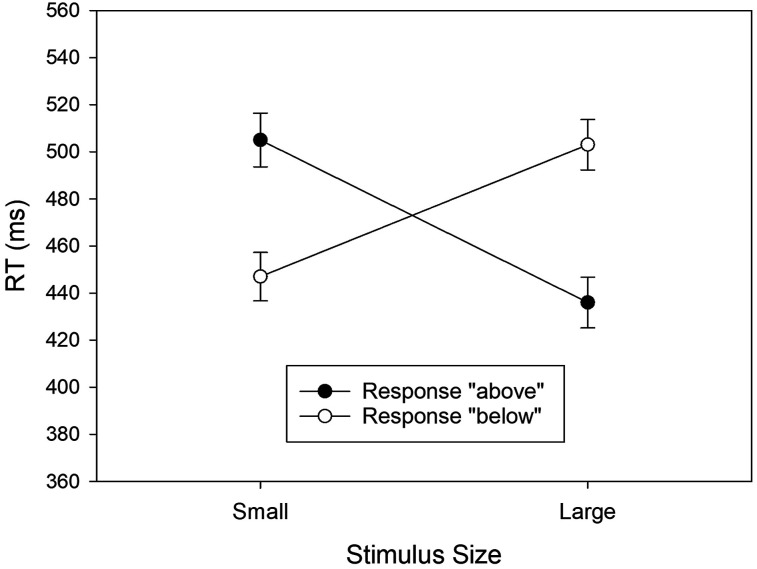
Mean RTs observed in Experiment 2 as a function of stimulus size and the vocal response. Error bars represent 95% confidence intervals for within-subjects designs (Cousineau, 2017).

We analyzed RTs from Experiment 2 in a two-factorial ANOVA with Stimulus Size and Response as within-subjects variables. The cell means from this design are shown in Figure 3. A significant main effect of Stimulus Size reflected shorter RTs to the large stimulus (*M* = 469 ms, *SD* = 81) relative to the small stimulus (*M* = 476 ms, *SD* = 79), *F*(1, 64) = 6.148, *MSE* = 493.758, *p* = .016, partial η^2^ = .088. The main effect of the Response was not significant, *F*(1, 64) = 2.862, *MSE* = 516.286, *p* = .096, partial η^2^ = .043. Most importantly, however, the two-way interaction was also significant, *F*(1, 64) = 87.028, *MSE* = 2,884.185, *p* < .001, partial η^2^ = .576. The interaction reflected faster “below” responses to small stimuli (*M* = 447 ms, *SD* = 65) relative to “above” responses (*M* = 505 ms, *SD* = 81), *t*(64) = −7.893, *p* < .001, *d* = −0.979, and faster “above” responses to large stimuli (*M* = 436 ms, *SD* = 64) relative to “below” responses (*M* = 503 ms, *SD* = 82), *t*(64) = 9.298, *p* < .001, *d* = 1.153.

#### Error Percentages

We analyzed error percentages from Experiment 2 in a two-factorial ANOVA with Stimulus Size and Response as within-subjects variables. The cell means from this design are reported in Table 2. The main effects of Stimulus Size, *F*(1, 64) = 0.004, *MSE* = 5.542, *p* = .948, partial η^2^ < .001, and Response, *F*(1, 64) = 0.135, *MSE* = 4.458, *p* = .715, partial η^2^ = .002, were not significant. However, the two-way interaction was significant, *F*(1, 64) = 36.814, *MSE* = 17.777, *p* < .001, partial η^2^ = .365. The two-interaction mirrored the RT results: “Below” responses were more accurate than “above” responses to small stimuli, *t*(64) = −5.924, *p* < .001, *d* = −0.735, and “above” responses were more accurate than “below” responses to large stimuli, *t*(64) = 5.078, *p* < .001, *d* = 0.630.

**Table 2:** Error percentages observed in Experiment 2 as a function of stimulus size and response

	Response	
	Below	Above
Stimulus size		
Small	0.846 (1.727)	3.923 (4.655)
Large	4.038 (5.334)	0.769 (1.871)
Standard deviations (*SD*) are given in parentheses.		

### Discussion

In Experiment 2, we observed a strong SSARC effect between the physical size of stimuli and the spoken responses “above” and “below.” The response “below” was faster and more often accurate to the small as compared to the large stimulus, and the response “above” was faster and more often accurate to the large as compared to the small stimulus. This result demonstrates that the vertical SSARC effect between physical stimulus size and vertical locations does not only occur when participants respond with their hands at different locations but also when the responses consist in spoken location words. This finding suggests that the vertical SSARC effect does not only reflect different sensorimotor experiences of the two hands but may also reflect more general, semantic knowledge as expressed in metaphors such as “more is up” (e.g., Lakoff, 1993).

## General Discussion

We investigated the existence and direction of associations between spatial size as a stimulus feature and vertical location as a response feature. In Experiment 1, participants had to press one of two vertically arranged keys to the size of a visual stimulus, and we independently varied the mapping between physical stimulus and response hand, as well as the mapping between physical stimulus size and vertical response location. We observed a hand-related SSARC effect: Left-hand responses were faster to the small relative to the large stimulus, whereas right-hand responses were faster to the large relative to the small stimulus. This observation replicates observations of a hand-related SSARC effect from studies in which the hands were located beside each other (e.g., Wühr & Seegelke, 2018; Wühr et al., 2024). In addition, we observed a location-related vertical SSARC effect: Responses at the lower location were faster to the small relative to the large stimulus, whereas responses at the higher location were faster to the large relative to the small stimulus. This is the first demonstration of a vertical association between stimulus size and response location that is independent from the effectors used for responding.

In Experiment 2, participants responded verbally to the physical size of a visual stimulus, and we again varied the S-R mapping. We observed a vertical SSARC effect in both the speed and accuracy of vocal responses: The “below” response was faster and more accurate to the small relative to the large stimulus, whereas the “above” response was faster and more accurate to the large relative to the small stimulus. Hence, the vertical SSARC effect can be observed both in manual and in vocal responses.

### Horizontal and Vertical Associations of Physical Size

In combination with previous findings, the present results show that the physical size of stimuli has effector-independent associations with both the horizontal spatial dimension and the vertical spatial dimension. The hand-independent relationship between physical stimulus size and the horizontal spatial dimension has been shown in studies in which authors varied the S-R mapping and the arm posture in a key-pressing task (Seegelke et al., 2023). One condition required participants to operate the keys with arms held parallel, whereas the other condition required participants to operate the keys with crossed arms. In the parallel-arms condition, the anatomical status of the (left vs. right) hand is aligned with relative key location (left vs. right key). However, in the crossed-arms condition, the anatomical status of the hand is in opposition to relative key location. If participants coded responses based on their hands, opposite mapping effects would be expected with different arm postures. However, if responses were coded based on key locations, similar mapping effects should occur regardless of arm posture (see also Wood et al., 2006).

Seegelke et al. (2023, Experiment 2) found only a regular SSARC effect with parallel arm posture, suggesting that participants in the manual key pressing task coded responses both in terms of hands and in terms of key (response) location, and both response codes contributed to the SSARC effect (cf. Wood et al., 2006, for a similar account of findings on the SNARC effect). With parallel arms, the stimuli activate hand and key-location codes on the same side, and these activations add up. In contrast, with crossed arms, the stimuli activate hand and key-location codes on different sides, and these activations compensate each other. Interestingly, task difficulty appears to influence the relative weights of hand codes and response-location codes. In a more difficult task, in which participants had to move levers to a target location, Seegelke et al. (2023) observed a reversal of the horizontal SSARC effect with crossed arms, suggesting a dominance of hand-based response coding.

The relationship between physical stimulus size and the *vertical* spatial dimension was first demonstrated by the significant size–location effect in Experiment 1. The orthogonal variation of hand-key relationships eliminated a possible influence of the hands on this effect. The results of Experiment 2 with vocal responses provided additional evidence for a hand-independent vertical SSARC effect. Hence, the horizontal SSARC effect (e.g., Wühr et al., 2024) and the vertical SSARC effect can be observed both with manual and with vocal responses. The SSARC effects in vocal responses suggest that these effects not only reflect sensorimotor habits but also reflect semantic knowledge that is expressed, for example, in linguistic metaphors (e.g., “more is up,” Lakoff, 1993).

### Possible Explanations for the Vertical SSARC Effect

There are, at least, two accounts of the horizontal SSARC effect, the polarity-correspondence principle and an experience-based account. Both accounts can also explain vertical SSARC effects. The polarity-correspondence principle assumes that participants ascribe polarities to stimuli and responses in many binary classification tasks (e.g., Proctor & Cho, 2006). To explain the vertical SSARC effect, we need to assume that physically small stimuli and the lower response location are coded as negative polarity, whereas physically large stimuli and the upper responses are coded as positive polarity (e.g., Richter & Wühr, 2022). The assumption of coding lower locations as negative and upper locations as positive polarity is supported by empirical evidence (e.g., Meier & Robinson, 2004; Proctor & Cho, 2006).

The polarity-correspondence principle is consistent with several characteristics of the SSARC effect. For example, the finding that relative stimulus size in a given stimulus set, and not absolute stimulus size, is responsible for the SSARC effect (Wühr & Richter, 2022) is consistent with polarity correspondence. There are, however, other findings that appear inconsistent with the polarity-correspondence principle. For example, we failed to observe the horizontal SSARC effect in a task in which response selection required participants to choose between two adjacent fingers of the same hand (Seegelke et al., 2023, Experiment 1). This finding contrasts with the effects of spatial compatibility that reliably occur in tasks in which response selection requires participants to choose between two fingers of the same hand (e.g., Heister et al., 1986, 1987). The spatial compatibility effects with choices between two fingers of the same hand strongly indicate that adjacent fingers are coded as “left” versus “right,” and therefore should also be assigned the corresponding polarities. Hence, the absence of horizontal SSARC effects with unimanual response sets suggest that polarity coding is not a crucial factor in producing the effect.

The CORE principle provides an alternative account of the SSARC effect (Pitt & Casasanto, 2020). To explain horizontal and vertical SSARC effects, CORE assumes that humans have experienced correlations between physical size and horizontal or vertical location, respectively, earlier in their lives. The existence of such correlations seems obvious. For example, the size of many real-world objects not only varies in the horizontal but also in the vertical direction. Therefore, one would assume stronger associations between “small” and “below” and between “large” and “above” than the other way round. This pattern of associations would reflect a “more is up” metaphor (Lakoff, 1993; Lakoff & Johnson, 1980). Most likely, these correlations are also learned through bodily experiences. When carrying a box with one hand below and one hand above the box, the upper hand is at a higher location when holding a large box than when holding a small box. Similarly, when piling up large objects, each object has to be lifted higher than when piling up small objects.

### Vertical SSARC Effects and Vertical SNARC Effects

The simultaneous observation of a hand-related SSARC effect and a location-related SSARC effect in Experiment 1 contrasts with the results of Wiemers et al. (2017a) on the SNARC effect. When attempting to explain the discrepant findings, we have to consider the preconditions for the occurrence of S-R compatibility effects.

Research has shown that the occurrence of S-R compatibility effects requires two preconditions. First, a critical response feature (e.g., anatomical hand status, response location) must be used for representing, and discriminating between, the possible responses at the response-selection stage (e.g., Ansorge & Wiihr, 2004; Wühr et al., 2008). This was obviously the case in both studies. In the present Experiment 1, the two responses differed both in the hand used for operating the key and in the relative spatial (i.e., vertical) location of the key. Because the two response features covaried, they provided redundant features for response representation and discrimination. Studies on spatial S-R compatibility effects with two-dimensional S-R arrangements have shown that compatibility effects can simultaneously occur for both spatial dimensions when responses covary on both dimensions, and thus, both spatial response features can be used for response representation (e.g., Ansorge & Wiihr, 2004; Wühr et al., 2008). Response hand and vertical location also covaried in the present Experiment 1, and therefore, both response features could be used for response representation, providing a basis for compatibility effects to occur.

The second precondition for S-R compatibility effects is called “element-level compatibility” (e.g., Fitts & Deininger, 1954; Kornblum et al., 1990), which denotes the existence of associations between elements of the stimulus set and elements of the response set. The hand-related SSARC effect (Experiment 1; Wühr & Seegelke, 2018), for example, suggests that physically small stimuli are more strongly associated with the left as compared to the right hand, whereas physically large stimuli are more strongly associated with the right as compared to the left hand. The location-based vertical SSARC effect suggests that physically small stimuli are more strongly associated with a lower relative to a higher response location, whereas physically large stimuli are more strongly associated with a higher relative to a lower response location. Similarly, the hand-related SNARC effect observed by Wiemers et al. 2017a, (Experiments 1 and 2) suggests that – in some populations, at least – small numbers are more strongly associated with the left as compared to the right hand, whereas large numbers are more strongly associated with the right as compared to the left hand.

Given the two preconditions, there are two possible accounts for the absence of the location-based vertical SNARC effect in Experiments 1 and 2 by Wiemers et al. (2017a). First, participants only used the hand feature, and not the vertical location feature, for representing and discriminating the possible responses in the two-choice task. Second, specific associations between different numbers and different vertical locations do not exist. The fact that Wiemers et al. (2017a) observed a location-based vertical SNARC effect in their Experiment 3, when the mapping between hands and response keys alternated between blocks, favors the first explanation. A question for future research is why participants used both response features (hand, location) for representing and discriminating responses in our Experiment 1, while the participants of Wiemers et al. (2017a) used only the hand feature for representing and discriminating responses in their Experiments 1 and 2.

### Conclusions

The present results suggest that the physical size of visual objects is not only associated with horizontal locations but also with vertical locations. These associations presumably reflect learned correlations between the size of objects and their extension in both the horizontal and the vertical direction. Moreover, the observation of a size–space compatibility effect in vocal responses may indicate that they do not only reflect sensorimotor experiences but also semantic knowledge, which is also expressed in linguistic metaphors.

## Footnotes

^1^
Walsh (2003) has coined the term Spatial-QUantity Association of Response code (SQUARC) effect as an umbrella term for different instances of compatibility effects between space and a quantitative (prothetic) variable such as, for example, numerical magnitude (SNARC effect), physical size (SSARC effect), or time (so-called STEARC effect; see Umiltà et al., 2018, for a short review).

^2^
We will call the effect of the mapping between a relevant stimulus feature (e.g., stimulus size) and response location a “compatibility” or “mapping” effect. In contrast, we will call the effect of the relationship between an irrelevant stimulus feature (e.g., stimulus size) and response location a “congruency” effect.

^3^
We altered the presentation duration of fixation points between 400 and 600 ms to prevent participants from responding in a constant rhythm and therefore to increase participants attention for the imperative stimulus.

^4^
We gave error feedback throughout the experiment to provide help if participants became unsure about the correct S-R mapping. This procedure has proven useful in previous studies.

^5^
The three-factorial design is different from, but equivalent to, the two-factorial design described in the pre-registration of Experiment 1. In our pre-registration, we had planned to combine the factors Stimulus Size and Response Location to the higher order factor Size-Location Mapping, and to combine the factors Response Hand and Response Location to the higher-order factor Hand-Location Assignment. However, we now consider it more informative to perform the analysis with the three basic factors.

^6^
We compared responses with the same hand to different stimuli because this comparison is less affected by the almost significant main effect of Response Hand, relative to comparing responses with different hands to the same stimulus.

^7^
Although our German participants responded with the German words “unten” (for “below”) and “oben” (for “above”), we use the English location words in the text for clarity.

## Notes

**Publication Ethics:** The local Ethics Committee at TU Dortmund University (2018-09) approved the experimental protocol for both experiments.

**Open Science:** The manuscript includes a DOI generated by a public data repository pointing to the raw data underlying the findings reported in the article.

Open Data: The data sets are available at the Mendeley repository (https://data.mendeley.com/datasets/8jw866px99/2; Wühr, 2025).

Open Materials: Materials are not available in full.

Pre-registration: Experiment 1 was pre-registered at OSF (https://doi.org/10.17605/OSF.IO/9NYQ7; Wühr, 2023a). The three-factorial design used for analyzing Experiment 1 is different from, but equivalent to, the two-factorial design described in the pre-registration of Experiment 1. In our pre-registration, we had planned to combine the factors Stimulus Size and Response Location to the higher order factor Size-Location Mapping and to combine the factors Response Hand and Response Location to the higher-order factor Hand-Location Assignment. However, we now consider it more informative to perform the analysis with the three basic factors. Experiment 2 was pre-registered at OSF (https://doi.org/10.17605/OSF.IO/HRWQM; Wühr, 2023b). The pre-registered experiment and analyses were executed exactly as registered.
